# A randomized controlled trial to evaluate an adapted community health worker-delivered breast and cervical cancer screening education and navigation intervention for underserved Latina adults

**DOI:** 10.3389/fonc.2026.1687113

**Published:** 2026-03-11

**Authors:** L. S. Savas, N. I. Heredia, Y. Huyan, S. Coan, S. Martin, D. S. Mantey, E. M. Adlparvar, K. G. Yao, A. Alaniz, E. Figueroa, M. E. Fernandez

**Affiliations:** 1 Department of Health Promotion and Behavioral Sciences, Center for Health Promotion and Prevention Research, The University of Texas Health Science Center at Houston, School of Public Health, Houston, TX, United States; 2 Department of Biostatistics and Data Science, The University of Texas Health Science Center at Houston, Houston, TX, United States; 3 Department of Health Promotion and Behavioral Sciences, Michael and Susan Dell Center for Healthy Living, School of Public Health, The University of Texas Health Science Center at Houston, Austin, TX, United States

**Keywords:** breast cancer screening, cervical cancer screening, community health worker, community-based research, intervention, Latina, navigation, randomized controlled trial

## Abstract

**Introduction:**

We evaluated the effectiveness of an adapted community-based breast and cervical screening behavioral intervention. Community health workers (CHW) delivered group education and optional telephone-based health coaching and navigation on mammography and Papanicolaou (Pap) test screening among low-income Latinas in the Greater Houston area.

**Methods:**

CHWs recruited women (40 years and older) who were non-adherent to mammography screening guidelines (n=797), as well as women 21 years and over who were non-adherent to cervical cancer screening guidelines (n=501). Data collectors completed baseline surveys, randomized women to the CHW-delivered intervention and comparison (delayed intervention) arms, and completed 6-month follow-up surveys.

**Results:**

At follow-up, 39.9% in the mammography intervention arm and 20.3% of women in the comparison arm completed mammograms (*P* < .001). For cervical cancer screening, 55.8% of women in the intervention arm and 27.4% of women in the comparison arm completed cervical screening (*P* < .001). Adjusting for socio-economic, demographic, and cancer screening history factors significant in bivariate analysis (*P* <.25), women in the mammography intervention had a significantly increased odds of receiving a mammogram based on adjusted intention-to-treat (aOR: 2.00, 95% CI: 1.40–2.84) and per-protocol analyses (aOR: 2.53, 95% CI: 1.74–3.68). Similarly, women in the Pap test screening intervention arm had a significantly increased odds of Pap test screening in intention-to-treat (aOR: 1.95, 95% CI: 1.29–2.95) and per-protocol analyses (aOR: 3.51, 95% CI: 2.18–5.65).

**Conclusion:**

This study provides strong evidence that community-based group education and navigation increase breast and cervical screening among low-income Latinas in urban settings. Future research to scale up this evidence-based CHW-delivered program is needed.

## 1 Introduction

Despite the established effectiveness of screening on reducing breast and cervical cancer morbidity and mortality, and reducing incidence of cervical cancer (1, 2), use of screening services remains suboptimal in the United States (U.S.), particularly among Latina/Hispanic women (hereafter Latina), uninsured and publicly insured women, and those living in low-income households (3–5). Women who underutilize breast and cervical cancer screening have a higher prevalence of late-stage cancer diagnoses due to missed opportunities for prevention (cervical), early detection, and treatment (6), resulting in lower survival (7). While Latinas are less likely to develop breast cancer compared to non-Hispanic White women (NHWs) (96.3 versus 132.5, per 100,000, respectively) (8), they are also less likely to be diagnosed at an early stage (8). The delay in screening contributes to lower breast cancer five-year survival rates compared with NHW women (88% vs. 92%, respectively) (9). Nationally, Latina adults have a higher lifetime probability of developing cervical cancer (1 in 115) compared with NHWs (1 in 180), slightly lower cervical screening prevalence rates (83% vs. 86%), and a higher probability of cervical cancer mortality (1 in 350) compared with NHWs (1 in 516) (8).

Cancer screening underutilization is a multifaceted problem for low-income women (5), particularly those affected by structural or environmental barriers that limit access, such as transportation (5, 10, 11), and economic barriers, including costs associated with screening (12–15), and not having health insurance (16, 17). Despite the availability of screening at U.S. safety net clinics, such as Federally Qualified Health Centers (FQHCs), where no or low-cost cancer screenings are available to economically disadvantaged groups, cancer screening is suboptimal in this population (18, 19). Other important screening determinants among uninsured, underinsured, or low-income adults include language barriers, lack of knowledge, and psychosocial barriers, such as perceived efficacy of cancer screenings, self-efficacy to access care, and self-efficacy to receive screenings (14, 20–27).

Currently (December 2025), the American Cancer Society (ACS) recommends that average-risk women 40–44 years receive the option to start annual mammogram screening, women 45–54 receive an annual mammogram, and women 55 years and older have an option to obtain an annual or every other year mammogram screening (9). For cervical screening, since the current study was conducted, the ACS updated recommendations, raising the age to begin screening from 21 to 25 years, and continuing screening through age 65 years. Cervical cancer screening options have also changed and include: 1) (preferred option) screening every five years through primary human papillomavirus (HPV) testing alone, 2) screening every five years through co-testing (both HPV testing and Papanicolaou (Pap) testing), and 3) screening every three years with Pap testing (28, 29). The ACS also continues to recommend catch-up HPV vaccination for adults through age 26 years (30). Changing guidelines makes the need for such community-based intervention studies even stronger.

The purpose of this study was to adapt and evaluate a community-based intervention, called *Cultivando la Salud* (CLS) program. The original program (CLS) engaged community health workers (CHWs) to deliver one-on-one education and provide referrals to local clinics to increase mammography and Pap test screening rates among Mexican American women living in farmworker communities. In a multi-site intervention study conducted in rural communities on the U.S.-Mexico border, the program increased mammography and Pap test screening based on per-protocol analysis (31). The program comprised evidence-based methods and strategies, such as peer role models, positive social reinforcement, and persuasive messaging, designed to address screening psychosocial determinants, such as self-efficacy to get a screening (32). To increase screening among culturally and geographically diverse low-income Latina adults in an urban setting, we adapted the CLS intervention (now called *Salud en Mis Manos* or *Health in My Hands*) and examined its effectiveness on increasing both mammography and Pap test screening among Latina adults in the metropolitan area in and around Houston, Texas.

## 2 Materials and methods

### 2.1 Study design

UTHealth Houston School of Public Health (SPH) research team partnered with a non-profit CHW organization, ProSalud, Inc. to adapt the CLS program for medically underserved Latina adults residing in Houston, Texas, community settings. We used a randomized controlled trial (RCT) design to examine the effectiveness of the adapted program on increasing mammography and Pap test screening (primary outcomes). We determined the sample size based on equal randomization of participants to the intervention and control groups. Expected effect sizes were based on effect size estimates from the original CLS study conducted by Fernandez et al. (31). For mammography screening, we powered the study to detect a 10.9-percentage point increase in mammography screening uptake (from 29.9% to 40.8%). With 80% power and a two-sided alpha level of 0.05, we estimated we needed a total of 602 participants. Anticipating 25% attrition, we planned to recruit 803 participants (approximately 402 assigned to each arm). For the cervical screening cohort (Pap test screening), we powered the study to detect a 15.9-percentage point increase in Pap test screening uptake (23.6% to 39.5%). Using the same power and alpha assumptions, we estimated that we needed a total of 266 participants. Anticipating 25% attrition, we aimed to recruit 356 participants (178 assigned to each arm). Participants completed baseline surveys (pre-test), were randomized to intervention or delayed comparison (control) arms, and follow-up assessments began 6 months post-baseline survey (see Data Collection section below).

### 2.2 Program adaptation

The adapted program, initially referred to as CLS-Houston and later renamed *Salud en Mis Manos* (SEMM), included three main components designed to increase breast and cervical cancer screening: 1) a bilingual CHW-led group education session, based on the original CLS curriculum (using a flipchart and optional video, which became outdated and dropped over time) to address psychosocial determinants of screening, 2) a clinic referral database developed for the new setting, used by CHWs to provide participants with tailored lists (based on residence) of local and affordable clinics where they could access recommended screenings, and 3) a new telephone-based health coaching and navigation protocol delivered by bilingual health coach navigators. The group education was one hour long and retained the same methods as the original CLS intervention, which comprised behavior change techniques, such as role models, testimonials, and persuasive communication to convey cancer screening messages targeting psychosocial determinants of screening, as well as to increase knowledge of screening guidelines (31, 33). The optional health coach navigation assistance included ongoing phone calls, delivered by trained health coaches (lay health workers or CHWs) to provide personalized support and to assist participants in overcoming complex barriers to screening.

Briefly, the UTHealth Houston/ProSalud program planning team (including CHWs, who were Latinas from the priority communities) collaborated to adapt the program for the new priority population and urban setting, the Greater Houston area. Due to new HPV vaccination recommendations for young women, HPV vaccination education was also included as a new behavioral outcome for women receiving the cervical cancer screening education (26 and younger) (34). The team rapidly adapted CLS using participatory methods, collaborating with CHWs from the priority community and with experience delivering other health education programs to Latina women in the program target area. To synthesize evidence and guide the adaptation process, we used the Intervention Mapping framework for adaptation (35). The adaptation process was theoretically grounded in Fishbein’s Integrated Behavioral Model (36) and informed by evidence from a literature review of previous breast and cervical screening interventions, including the original CLS evaluation (31) and an intervention research study focused on barriers to Pap test screening, conducted in the same priority community (20, 37).

After identifying important screening determinants, behavior change methods, and implementation strategies, the adaptation team reviewed program materials and potential personal-level and environmental barriers to screening to identify areas for modification. Minor changes to the education materials reflected updated American Cancer Society (ACS) screening guidelines and surface adaptations to represent the urban setting and younger women included in the adapted program. Largely informed by the CHWs’ experiences accessing clinical services, as well as their experiences helping uninsured and underinsured women overcome complex structural barriers frequently encountered in urban settings, the team developed a navigation protocol to provide ongoing support to address psychosocial barriers, as well as assistance to overcome structural barriers to completing clinical services.

The original CLS program was developed for Mexican American women living in farmworker communities, primarily in rural settings, and the educational program was delivered door-to-door in participants’ homes. To address barriers to door-to-door recruitment and intervention delivery in an urban setting, the UTHealth-ProSalud adaptation and implementation planning team co-designed a new community outreach strategy to identify, enroll, and deliver the program to participants in community settings. This change was in response to CHWs’ safety concerns regarding recruitment in residential settings and delivery of in-person education in participants’ homes. The planning team also decided to focus on providing group education sessions, coordinating with community centers and other organizations to plan sessions in public spaces embedded in the priority communities. The new focus on group education required adapting the CHW training to include behavior change techniques and group facilitation techniques required to deliver group education sessions. CHWs were trained to prompt discussions to address confusion about screening guidelines and to facilitate discussions to address beliefs and attitudes that emerge during discussions. Group education sessions also created opportunities for CHWs to role model different strategies for overcoming barriers, followed by group discussions (e.g., sharing personal testimonials of how they overcame similar barriers). CHWs also learned to facilitate peer support and encourage participants to share positive experiences as relatable examples, leveraging opportunities for participants to serve as each other’s role models to overcome screening barriers.

The health coach navigators (not necessarily certified CHWs) were trained to compile the clinic referral database for uninsured and underinsured women, and to understand and provide one-on-one assistance to address personal barriers and connect participants with clinics to complete their mammogram and Pap test screenings. Navigators used accessible information on the internet and provided during training to identify safety net clinics, such as FQHC listings. They called clinics to compile relevant information to provide useful clinic referrals to participants, such as location, clinic hours, and information about requirements to qualify for low-cost services. They maintained the database to ensure referrals were accurate to best assist participants in accessing convenient and affordable care. Together, these strategies aimed to address knowledge and behavioral determinants, as well as structural barriers to cancer screenings (e.g., identifying clinics, making appointments, or planning transportation).

*Community outreach.* To support community outreach and community-based delivery of the education session to groups, the program planning team identified neighborhoods to prioritize and developed community engagement plans. Recruitment efforts were concentrated in Greater Houston communities with high proportions of low-income Latina adults. ProSalud CHWs and leadership, and the UTHealth Houston SPH program team, networked with local organizations serving the priority Latina adult community to garner support from local leaders, community centers, social service organizations, and small businesses. To increase community awareness about the program, the team members posted flyers in community settings, such as food pantries and laundry mats, and CHWs and project staff disseminated information about the program directly to Latina women, reaching them in places frequented by the priority community, such as back-to-school events, health fairs, English as a Second Language, Head Start, and neighborhood grocery stores. Project staff also promoted the program on Spanish radio and television stations. Community engagement resources included print materials and packets for community leaders, developed to provide information about the program goals and different options for community partners to support the effort (e.g., promotional materials). Social marketing resources were developed to promote awareness of the program, such as flyers, posters, and short video promotions that played in community centers. Community organizations that partnered with the program provided CHWs with space to recruit and deliver the program. The three-year project, including adaptation of the project, was conducted 2011–2014, and the UTHealth Houston Committee for the Protection of Human Subjects approved the research protocol (IRB# HSC-SPH-11-0179). This study was registered in ClinicalTrials.gov (NCT04426019).

### 2.3 Participants

Study participants were Latina adults residing in the Greater Houston area non-adherent to mammography and/or Pap test screening. We defined screening need according to ACS guidelines at the time of the study and included women aged 21–64 years and older who had no Pap test screening in the previous three years, and women 40 years and older with no mammography screening within the previous two years (38). Thus, we lowered the original program inclusion age from 50 years to 40 (for the breast screening cohort) and from 50 years to 21 years (for the cervical screening cohort). Spanish*-*speaking CHWs and bilingual UTHealth Houston research staff used the program breast and cervical cancer screening need assessment form to determine eligibility based on women’s self-reported age, self-identified Hispanic/Latino ethnicity, cancer history (none), mammography history (never screened or no previous mammography screening in the previous two years), Pap test screening history (never screened or no Pap test screening in the previous three years), no hysterectomy for the Pap test screening cohort, and willingness to provide telephone contact information. CHWs or bilingual staff recruiters explained the program and purpose of the program evaluation as part of the study consenting procedures. Women who needed at least one screening test (mammography or Pap test screening) were consented and enrolled in the program. Women could belong to one or both screening outcome cohorts, depending on their eligibility and screening need(s).

### 2.4 Data collection

After CHWs identified and consented eligible women, UTHealth Houston bilingual data collectors contacted women to complete the baseline survey, and six months later, to complete follow-up surveys. After completing baseline surveys, data collectors used the computer-generated randomized number to assign women to either the intervention or comparison arm (delayed intervention arm). Data collectors were blinded to the allocation process; however, following randomization, participants, data collectors, and intervention staff were aware of group assignment. After completing the baseline survey and allocation to study arms, intervention participants were scheduled to attend the one-hour CHW-delivered education session, and women in the comparison (delayed intervention) arm were provided print materials on cancer screening guidelines. After completing the follow-up survey, data collectors invited comparison arm participants to attend the CHW-delivered education session and navigation program. For each survey, participants received a $20 gift card in compensation for their time.

### 2.5 Measures

#### 2.5.1 Primary outcomes

The two primary outcomes, mammography screening and Pap test screening, were determined by self-reported answers to the follow-up survey questions for women enrolled in the mammography study cohort: *Since the last interview, about 6 months ago, have you had a mammogram?* And *What was the month and year of your most recent mammogram?* To determine Pap test screening outcomes, women enrolled in the Pap test screening cohort were asked: *Since the last interview, about 6 months ago, have you had a Pap test?* And *What was the month and year of your most recent Pap test?* to ensure screening happened after enrollment in the study. Concordance, sensitivity, and specificity of Latinas’ self-reported breast and cervical cancer screening have been evaluated in previous research (31), and found to have good agreement according to Tisnado’s criteria (39).

#### 2.5.2 Sociodemographic and psychosocial covariate measures

Baseline surveys included the following: age as a continuous measure, marital status (married, yes or no), family household income categorical measure based on the Health Information National Trends Survey (40) (9 categories, ranging from none to $75,000 and over), categorical health insurance status (none, private, Medicare, Medicaid, Veterans’ military insurance, State or local insurance, or other), and acculturation measured using the Marin Short Acculturation Scale for Hispanics (SASH; low vs high or bi-cultural) (41). This 4-item acculturation scale retained predictive value, validity, and reliability of the original 12-item scale (42). The four questions that comprise the scale include: 1) In general, what language(s) do you read and speak?, 2) What language do you usually speak at home?, 3) In what language do you usually think?, 4) What language do you usually speak with your friends?. Responses were on a five-point scale: 1 being “Only Spanish and 5 being “Only English. Summary scores were calculated for each respondent based on the mean of their responses to all four items. The resulting symmetric acculturation score was further dichotomized, with values <3 classified as low acculturation and values ≥3 classified as high or bicultural acculturation. In addition, we included years of education (continuous), born in Mexico (yes or no), family history of breast cancer (yes or no), and family history of cervical cancer (yes or no). Both the baseline and six-month follow-up surveys included psychosocial constructs operationalized using 5-point Likert-type scales, based on the original 2009 CLS trial scales (31, 43). We used one item to assess social norms for getting a mammogram (i.e., Other women like you get mammograms). We used the full self-efficacy 8-item scale measuring confidence to obtain cervical cancer screening (original Cronbach’s alpha = .95; current study alpha = .82). We also shortened and examined internal consistency using Cronbach’s alpha for the following scales: a 6-item scale measuring self-efficacy for mammography screening (alpha = .71); a 3-item scale assessing the pros (benefits) of mammography (alpha = .82); and 4-item scales assessing subjective norms for mammography (alpha = .71) and Pap testing (alpha = .64). Items that comprise psychosocial measures are presented in **Tables 1A, 1B**. Data were collected using computer-assisted survey software (QDS™). Surveys took 20–30 minutes to complete (for women due for one screening), 30–45 minutes for women who needed both breast and cervical screenings).

**Table 1A:** Pap test screening psychosocial measures, items, and internal consistency reliability.

Pap Testing Measures	Items	Cronbach’s Alpha
Knowledge 0 to 6 Number of correct answers among 6 questions	About how often do you think a healthy woman your age should get a Pap test? Women who do not have regular Pap screenings are more likely to have advanced cervical cancer when they were diagnosed. [yes] I need a Pap test only when I experience problems like pain or vaginal bleeding that is not my period. [no] A Pap test can detect problems before they become cancer. [yes] A lack of hygiene can cause cervical cancer. [no] Human Papillomavirus (HPV) can lead to cervical cancer. [yes]	NA
Self-efficacy 1 to 5 (Very unsure to very sure) Symmetric variable of 8 questions	How sure are you that you can discuss having a Pap test with your health care provider even if (s)he does not bring it up? How sure are you that you can schedule a Pap test appointment? How sure are you that you can keep having Pap tests, even if you had to go to a new office to get one? How sure are you that you can ask your primary care physician (doctor) for a referral to get a Pap test? How sure are you that you can go to get your next Pap test? How sure are you that you can get a Pap test even if you are worried that it will be painful? How sure are you that you can get a Pap test even if a friend discouraged you from having one? How sure are you that you can get a Pap test even if you had to pay for it?	0.82
Subjective Norms 1 to 5 (Strongly Disagree to Strongly Agree) Symmetric variable of 4 questions	Your family thinks that you should go get a Pap test. You want to do what your family thinks you should do about a Pap test. The doctor or health professional thinks you should be screened for cervical cancer. You want to do what the doctor or health professional thinks you should do about a Pap test.	0.64

**Table 1B:** Mammography screening psychosocial measures, items, and internal consistency reliability.

Mammography Testing Measures	Items	Cronbach’s Alpha
Knowledge 1 or 0 (Correct or Wrong)	“How often do you think a healthy woman your age should get a mammogram?”	NA
Self-efficacy 1 to 5 (Very Unsure to Very Sure) Symmetric variable of 5 questions	How sure are you that you can discuss having a mammogram with doctor even if (s)he does not bring it up? How sure are you that you can ask your doctor for a referral to get a mammogram? How sure are you that you can get a mammogram even if you are worried that it will be painful? How sure are you that you can get a mammogram even if you examined your own breasts and nothing seemed wrong? How sure are you that you can get a mammogram even if you had to pay for it?	0.71
Subjective Norms 1 to 5 (Strongly Disagree to Strongly Agree) Symmetric variable of 4 questions	Your family thinks you should have a mammogram. You want to do what your family thinks you should do about a mammogram. The doctor thinks you should get a mammogram. You want to do what the doctor thinks you should do about a mammogram.	0.71
Perceived Pros (benefits) 1 to 5 (Strongly Disagree to Strongly Agree) Symmetric variable of 4 questions	Having a yearly mammogram will give me a feeling of control over my health. Regular mammograms give you peace of mind about your health. Mammograms are necessary even when there is no history of breast problems in a family. Any discomfort from a mammogram is worth the benefit I get.	0.82
Social Norms 1 to 5 (Strongly Disagree to Strongly Agree)	“Other women like you get mammograms.”	NA

### 2.6 Data analysis

For both the mammography and Pap test screening cohorts, we examined the baseline frequency distributions for all categorical variables and descriptive statistics for continuous variables, including range, mean, and standard deviation. To measure differences between the intervention and comparison arms within screening cohorts, we used two-tailed t-tests for continuous variables, such as age and education, and chi-square tests for categorical variables, such as screening history and other health-seeking behaviors (44). After conducting descriptive statistics, we conducted a series of bivariate logistic regression analyses comparing baseline treatment condition and baseline demographic variables to identify systematic differences that should be adjusted for as covariates in our final models. For covariate selection, we used a more conservative statistical significance threshold (*P* ≤.25) (44–46) to identify covariates to include in the multivariable analyses for each outcome cohort. Using more liberal thresholds during initial screening helps to avoid excluding covariates that may later function as important confounders once other predictors are included (44). We also used purposeful selection to ensure meaningful covariates were included (e.g., age). We conducted intention-to-treat (ITT) and per protocol (PP) analyses to examine the intervention effect for each outcome, mammography, and Pap test screening. ITT analysis included all participants as originally randomized and who completed a baseline survey, regardless of participants’ retention and completion of the follow-up survey. The PP analyses included those who completed both the baseline and follow-up surveys.

As a secondary analysis, we examined the psychosocial and knowledge screening constructs targeted by the intervention. We computed mean scale scores for each construct and assessed differences across intervention and comparison arms at follow-up using linear regression. For each regression analysis, we adjusted for the construct’s baseline survey score and demographic factors significantly related to outcomes (at the *P* <.25). Analysis was completed using StataNow 19.5.

## 3 Results

### 3.1 Descriptive characteristics

**Figure 1:**
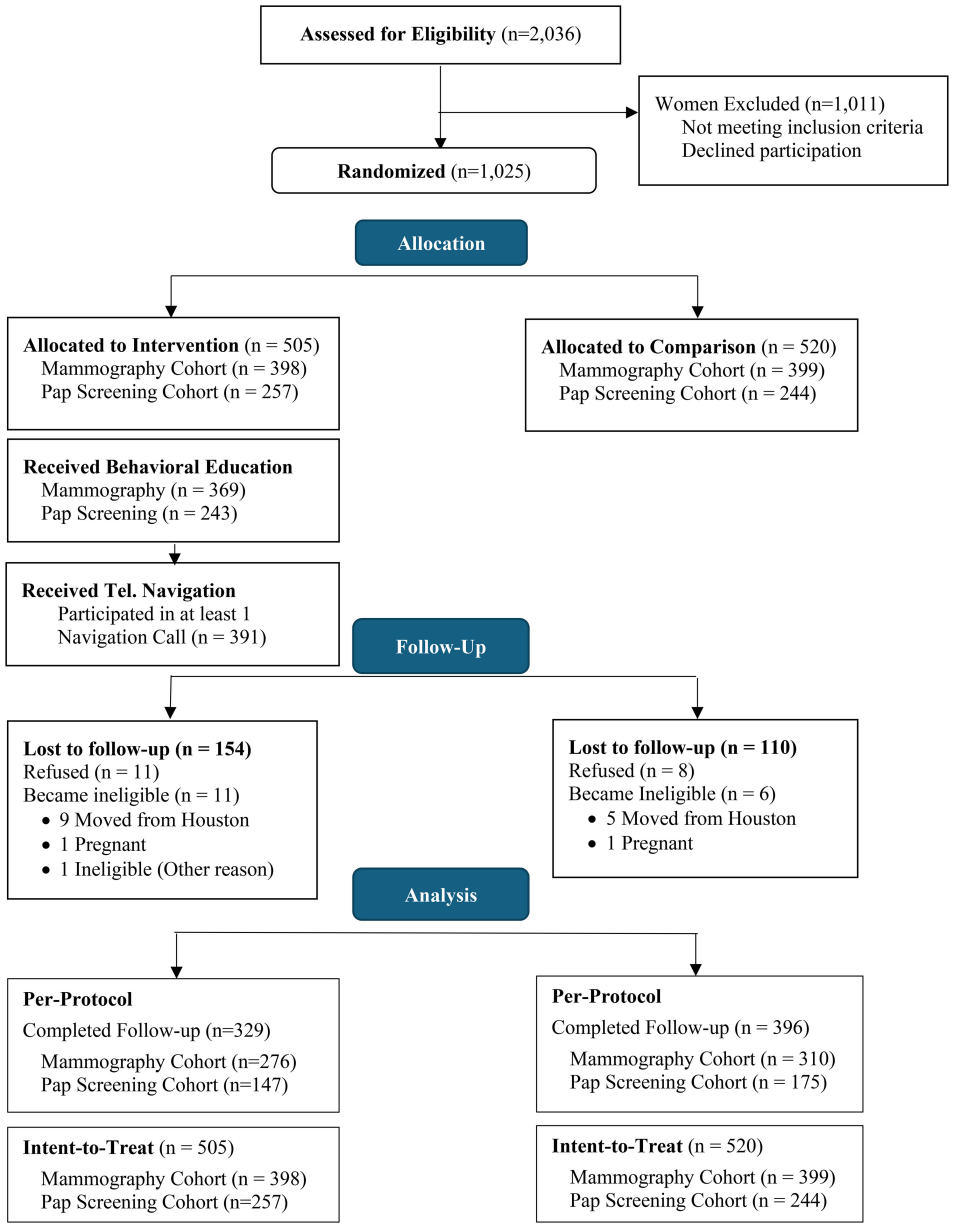
Community-based CHW-delivered intervention study sample: Consolidated Standards of Reporting Trials (CONSORT) Diagram. Note: The total mammography and Pap test screening cohorts, within each intervention and comparison group, do not equal group totals due to overlap between screening cohorts.

Among 2,036 potential participants who completed the brief breast and cervical cancer needs assessment, 1,025 were eligible and consented. Most women completed baseline surveys by telephone (98.9%), rather than in person (1.2%). Because some participants were eligible for both mammogram and Pap test screenings, the two samples overlapped. As a result, fewer total women were recruited than initially projected. However, more women in the mammogram group also needed a cervical screening than originally expected, increasing the Pap screening baseline sample relative to expectations. Despite higher-than-anticipated attrition overall, the final analytic sample was closer to the required sample size for the cervical screening outcome cohort, and lower for the mammogram screening outcome cohort (see Consort diagram). As shown in the Consort diagram (**Figure 1**), after completing baseline surveys, 505 were randomized to the intervention arm and completed the education session, and 520 were randomized to the comparison arm and received usual care (print education materials). Among women identified as non-adherent to mammography (n=797) or Pap test screening (501), 273 needed both mammogram and Pap test screening, 524 needed a mammogram only, 200 needed a Pap screening only, and 28 needed a Pap screening and HPV vaccine.

Bivariate analyses results indicated no difference in demographic, socioeconomic, and screening history factors between the intervention and comparison arms at baseline (**Table 2**). Women in both the mammography and Pap test screening cohorts had mean ages of 48 and 42 years of age, respectively. Most reported annual household incomes of less than $15,000 (56.9% in the mammography cohort and 53.5% in the Pap cohort), having no health insurance (95.2% in the mammography cohort and 95.5% in the Pap cohort), and low acculturation according to the Marin Acculturation Short Scale (94.2% in the mammography cohort and 93.4% in the Pap cohort). Within the mammography cohort and Pap test screening cohorts, we also compared the psychosocial constructs’ baseline mean scores across intervention and comparison arms using t-tests and found no significant differences between intervention and comparison arms in the mammography cohort. However, within the Pap test screening cohort, participants’ self-efficacy mean scores were significantly higher in the intervention arm versus the comparison arm [mean (SD): 4.42 (0.59) vs. 4.27 (0.75); *P* = .014].

**Table 2:** Baseline characteristics of women in mammography and pap test screening cohorts.

Characteristics	Mammography cohort (n=797)		Pap cohort (n=501)	
	Intervention (N = 398)	Comparison (N = 399)	Intervention (N = 257)	Comparison (N = 244)
Age (mean ± SD)	48.8 ± 8.1	48.1 ± 7.6	42.4 ± 10.4	41.5 ± 10.6
Education years (mean ± SD)	8.3 ± 4.6	8.2 ± 4.3	8.6 ± 4.2	8.4 ± 4.0
	n (%)		n (%)	
Marital status				
Never married	70 (17.6)	72 (18.1)	48 (18.7)	40 (16.4)
Married or living together	227 (57.0)	228 (57.1)	153 (59.8)	158 (64.8)
Divorced, separated, widowed	101 (25.4)	99 (24.8)	55 (21.5)	46 (18.9)
Income				
<$15,000	157 (54.1)	176 (59.7)	96 (51.1)	101 (56.1)
≥$15,000	133 (45.9)	119 (40.3)	92 (48.9)	79 (43.9)
Health insurance coverage				
No health insurance	381 (95.7)	378 (94.7)	242 (94.2)	234 (96.7)
Has health insurance	17 (4.3)	21 (5.3)	15 (5.8)	8 (3.3)
Family history of breast cancer				
Yes	50 (12.6)	65 (16.3)	30 (11.7)	42 (17.2)
No	346 (87.4)	333 (83.7)	227 (88.3)	202 (82.8)
Family history of cervical cancer				
Yes	66 (16.8)	68 (17.2)	44 (17.3)	46 (19.0)
No	326 (83.2)	328 (82.8)	211 (82.7)	196 (81.0)
Ever had a mammogram (Mammography cohort only)				
Yes	217 (54.5)	204 (51.1)		
No	181 (45.5)	195 (48.9)		
Ever had a Pap test (Pap Cohort Only)				
Yes			225 (90.4)	207 (89.22)
No			24 (9.6)	25 (10.8)
Acculturation (range 1-5)				
Low (<3)	375 (94.2)	381 (95.5)	240 (93.4)	227 (93.0)
High/bi-acculturation (≥3)	23 (5.8)	18 (4.5)	17 (6.6)	17 (7.0)
Country of origin				
U.S.	14 (3.5)	19 (4.8)	15 (5.8)	21 (8.6)
Mexico	227 (57.0)	242 (60.7)	164 (63.8)	161 (66.0)
Central America	130 (32.7)	112 (28.1)	65 (25.3)	53 (21.7)
Other non-U.S.	27 (6.8)	26 (6.5)	13 (5.1)	9 (3.7)

Note: No significant differences (*P*-value *<*.05) in demographic characteristics between intervention and comparison arms were detected in either cohort using t-tests (age, education) or chi-square tests for categorical variables.

Following completion of the baseline survey and allocation to study arms, among those in the intervention arms, 93% of women in the mammography screening cohort and 95% of women in the Pap test screening cohort participated in the CHW-delivered intervention education session, and 77% opted to receive at least one health coach navigation call. Follow-up survey completion rates were significantly lower in the intervention compared with the comparison arm for both mammography (69.35% vs 77.7%, respectively) and Pap test screenings (57.2% vs. 71.7%, respectively). We compared baseline demographics between the participants who completed follow-up surveys versus those lost-to-follow-up to assess differential attrition and found the groups were comparable, except mean level of acculturation was slightly lower among participants in the mammography final cohort [Mean (SD) score: 1.26 (0.61) participant vs. 1.37 (0.83) lost to-follow-up group; *P* = .047] as well as lower in the Pap test screening final cohort [Mean (SD) score: 1.31 (0.66) participant vs. 1.48 (0.89 lost to follow-up group; *P* = .017]. Women had a mean follow-up time from baseline to follow-up survey of 9.8 months, with the intervention arm having an average of 10 months versus 9 months for the comparison arm.

### 3.2 Program effectiveness: intention to-treat and per-protocol analyses of mammography and cervical screening outcomes

ITT and PP analyses were used to assess the effects of the intervention on mammogram and Pap test screening completion outcomes. A significantly greater proportion of women in the intervention arm within the mammography cohort received a mammogram (**Table 3**). Based on ITT analyses, 27.6% of women completed a mammogram compared with 15.8% in the comparison arm. Similarly, a significantly greater proportion of women in the intervention arm within the cervical screening cohort completed a Pap test. Based on ITT analyses, 31.9% completed a Pap test in the intervention arm compared with 19.7% in the comparison arm (**Table 3**). A statistically significant increase in mammography and Pap test screening remained when considering differences in time to completion of follow-up surveys.

**Table 3:** Unadjusted effectiveness of mammography and pap test screening: intention-to-treat* and per-protocol** .

Study arm	Intervention	Comparison	*P*-value
Mammography cohort	Completed Screening		
Intention-to-treat*	110/398 (27.6%)	63/399 (15.8%)	*P* <.001
Per-protocol**	110/276 (39.9%)	63/310 (20.3%)	*P* <.001
Pap cohort	Completed Screening		
Intention-to-treat*	82/257 (31.9%)	48/244 (19.7%)	*P* = .002
Per-protocol**	82/147 (55.8%)	48/175 (27.4%)	*P* <.001

*Intention-to-treat analyses of screening completion include all participants, regardless of follow-up, with the assumption that those with no follow-up survey had not been screened.

**Per-protocol analyses of screening include participants who completed the follow-up survey.

We also assessed the intervention effects in additional ITT and per-protocol PP multivariable models, adjusting for socio-demographic factors significant in univariable analyses at the *P* <.25 level. Within the mammography cohort women in the intervention arm had a significantly increased odds of receiving a mammogram compared with the comparison arm (adjusted OR: 2.00, 95% CI: 1.40–2.84), adjusting for age, level of education attainment, insurance status, county of origin (born in Mexico versus other), and mammography history (ever versus never). Educational attainment and mammography history remained significant in both ITT and PP analyses (**Table 4**). Within the Pap cohort, while acculturation and insurance status were identified as significant in PP bivariate analysis at *P* <.25, no factors were identified as significant in bivariate analysis among the ITT sample. However, following the purposeful selection of covariates approach, age, insurance status, and level of acculturation were included as covariates in the ITT model. Based on the Pap test screening ITT analysis, women in the intervention arm had a significantly increased odds of Pap test screening (adjusted OR: 1.95, 95% CI: 1.29–2.95). No covariates were significantly related to increased odds of Pap test screening in the final adjusted ITT model. When examining screening outcomes using PP analysis (i.e., limited to participants who complete the follow-up survey), the odds of both mammography and Pap test screening outcomes increased compared with ITT results (**Table 4**).

**Table 4:** Intervention effectiveness: adjusted logistic regression analyses.

Mammography screening*							
Intention-to-treat (n = 795)				Per-protocol (n = 583)			
Variables	aOdds ratio	*P*-value	95% C.I.	Variables	aOdds ratio	*P*-value	95% C.I.
**Intervention**	**2.00**	**<.001**	**(1.40–2.84)**	Intervention	**2.53**	**<.001**	**(1.74–3.68)**
Age in years	1.00	.70	(0.98–1.03)	Age in years	1.01	.70	(0.98–1.03)
Insurance (vs. No)	1.55	.25	(0.74–3.28)	Insurance (vs. No)	2.01	.11	(0.85–4.74)
**Years in school**	**1.05**	**.02**	**(1.01–1.10)**	**Years in school**	**1.05**	**.04**	**(1.00–1.09)**
Born in Mexico (vs. Other)	0.76	.13	(0.54–1.09)	Born in Mexico (vs. Other)	0.79	.22	(0.54–1.15)
**Ever had a mammogram** **(vs. Never)**	**1.59**	**.02**	**(1.08–2.34)**	**Ever had a mammogram (vs. Never)**	**1.53**	**.046**	**(1.01–2.32)**
Pap test screening*							
Intention-to-treat (n = 499)				Per-protocol (n = 320)			
Variables	aOdds Ratio	*P*-value	95% C.I.	Variables	aOdds Ratio	*P*-value	95% C.I.
**Intervention**	**1.95**	**.002**	**(1.29–2.95)**	**Intervention**	**3.51**	**<.001**	**(2.18–5.65)**
Insurance (vs. No)	0.44	.20	(0.12–1.56)	Insurance (vs. No)	0.47	.29	(0.12–1.89)
Age in years	0.99	.35	(0.97–1.01)	**Age in years**	**0.97**	**.03**	**(0.95–0.99)**
Acculturation High (vs. Low)	0.55	.24	(0.20–1.48)	Acculturation High (vs. Low)	0.99	1.00	(0.31–3.23)

*Mammography and Pap test screening ITT & PP analyses models adjusted for factors identified significant in bivariate analyses at *P <*.25.

Note: Significant results bolded.

### 3.3 Secondary analysis of psychosocial factors at follow-up

We compared mean scores of psychosocial factors at follow-up across the intervention and comparison arms to assess the potential effect of the intervention on these constructs. Because bivariate comparisons between intervention and comparison arms indicated no significant differences (based on *P-*value <.05) in demographic, socioeconomic, and cancer screening history (**Table 2**), we did not adjust for these factors in the analysis. We also examined baseline differences in psychosocial factors across study arms and only identified a significantly higher mean score for Pap test screening self-efficacy in the intervention versus comparison arm at baseline. Thus, when comparing Pap test screening self-efficacy scores across intervention and comparison arms at follow-up, we adjusted for baseline self-efficacy. **Table 5** presents comparisons of mean follow-up scores across study arms. Within both the mammography and Pap test screening cohorts, we found knowledge to be significantly higher in the comparison versus the intervention arm, while mammography and Pap test screening self-efficacy scores were higher in the intervention arms; the difference was only significant in the mammography cohort (**Table 5**).

**Table 5:** Psychosocial constructs measured at follow-up, by study arm.

	Intervention arm mean (95% CI)	Comparison arm mean (95% CI)	P-value
Mammography Cohort			
Knowledge*	**0.43 (0.38–0.48)**	**0.53 (0.48, 0.58)**	**.005**
Self-efficacy	**4.53 (4.44–4.61)**	**4.40 (4.32–4.48)**	**.042**
Perceived pros	4.57 (4.51–4.63)	4.50 (4.45–4.55)	.092
Social norms‡	**4.01 (3.89–4.14)**	**4.18 (4.07–4.30)**	**.048**
Subjective norms	4.13 (4.04–4.22)	4.14 (4.06–4.22)	.854
Pap test Screening Cohort			
Knowledge (range = 0–6)**	**1.76 (1.53–1.98)**	**2.20 (1.98–2.43)**	**.006**
Self-efficacy†	4.53 (4.42– 4.65)	4.45 (4.35–4.55)	.278
Subjective norms	4.23 (4.13–4.34)	4.12 (4.02–4.21)	.113

All scale scores except knowledge scales range from 1 to 5.

* Participants answered one mammography-related knowledge question.

†Adjusted for baseline self-efficacy.

‡Social norms is a single item measure.

** Composite score based on six Pap-related knowledge questions.

Note: Significant results bolded.

## 4 Discussion

Findings from this randomized controlled trial indicate that the community-based program adaptations for CHW-delivered group education, followed by the addition of a protocolized health coach navigation component, resulted in significantly increased mammography and cervical cancer screening outcomes in both the PP and ITT analyses. These results provide strong evidence of effectiveness for the adapted program (later renamed *Salud en Mis Mano, SEMM)*, replicating the original CLS study evidence based on PP analysis, and contributing new evidence of effectiveness based on ITT analysis (31). Furthermore, the present study demonstrates that the adapted intervention for younger women residing in urban settings was effective. While a similar adaptation of the CLS program replicated effectiveness for mammography in an urban setting in El Paso, the El Paso intervention included one-on-one education rather than group delivery and did not observe a change in cervical cancer screening among women under 50 years of age (47). Our findings fill an evidence gap by demonstrating that combining breast and cervical screening behavioral education in delivery to groups of low-income, and primarily uninsured women (due for one or both screenings) can effectively increase screening for both outcomes. This approach helps support CHW-delivered programs in community settings to groups of medically underserved women with varying screening needs through a single education session paired with offering telephone-based coaching and navigation support to address participants’ personal and structural screening barriers.

Community-based CHW-delivered intervention studies to increase breast and cervical cancer screening among low-income Latina adults have worked in rural settings to increase mammography (48), Pap test screening (49) or both (31, 37, 50, 51), while similar breast and cervical screening interventions among Latina adults in urban settings have mixed results (47, 52, 53). In a 2020 systematic review of community-based programs engaging CHWs to increase cancer screenings, seven studies included a comparison arm (54). Three of these studies focused on Latina adults (49, 52, 55), and one of these included group delivery and provided navigation sessions following education (55). These studies reported increased breast and cervical screening based on PP analysis only. A 2023 systematic review (51) of CHW-delivered cancer screening interventions identified three interventions that evaluated bundled breast and cervical screening using randomized trial designs reported results based in urban settings (47, 52, 56). One of these examined effectiveness using ITT analysis, however this intervention was delivered one-on-one and did not comprise groups of women with different screening needs (47). The present study adds to this evidence, demonstrating effectiveness of delivering bundled mammogram and Pap test screening behavioral group education in community settings, followed by telephone-based navigation, based on ITT and PP analyses. Additionally, our study findings indicated high intervention participation rates in both the education session and telephone-based navigation, demonstrating feasibility and strong program acceptance among medically underserved Latina adults. Specifically, among women identified in community settings with a screening need and enrolled in the adapted CLS (SEMM) program, about 95% attended their scheduled education session (**Figure 1**), which they had to make time to attend after the baseline survey was completed by telephone.

Our examination of the post-intervention intermediary psychosocial factors produced mixed results (**Table 5**). In both the mammography and cervical screening cohorts, at follow-up, the comparison arm participants had higher knowledge scores in both mammography and Pap test screening cohorts, which may reflect that the measures did not accurately detect a change in knowledge, or that while knowledge is necessary, it is not a main determinant of screening. Study procedures (survey, informal conversations with data collectors) likely had an impact on knowledge in both arms. However, what was more important in influencing behavior were other screening determinants that were influenced through the intervention. Higher knowledge scores in the comparison groups suggest that the CHW-delivered behavioral education and health coach navigation intervention components increased other important screening determinants, which led to intervention impact (rather than knowledge). The intervention likely helped participants overcome psychosocial and access barriers to complete their needed screenings. Additionally, in both the mammography and cervical screening cohorts, the mean self-efficacy score for obtaining screening was higher in the intervention group than in the comparison group, although the difference was not statistically significant in the Pap screening cohort. These results are similar to the original CLS trial findings (31). These findings support that the behavioral change methods embedded in the education and navigation protocol (e.g., role modeling and sharing testimonials about how other women like them overcame barriers and navigators’ facilitation of actions required to make and attend appointments) effectively increased self-efficacy. While results indicate more work is needed to strengthen the intervention to positively change other psychosocial factors examined, the strong evidence of intervention effectiveness on increasing screening underscores that this intervention’s success is strongly grounded in increasing self-efficacy, a focus of both the behavioral education and health coaching navigation approach.

While not the primary aim of this study, our results provide insight into the psychometric properties of our assessment of mammography perceived benefits, as well as self-efficacy, and subjective norms related to mammography and Pap test screening among low-income Latinas in Texas. These shortened scales’ internal consistency scores remain acceptable and comparable to the longer versions (31, 43). For example, our assessment of subjective norms used 4-items while the original scale used 6-items; nevertheless, the scales produced lower alpha scores for mammography (.71 for 4-items; .80 for 6-items) and Pap test screening (.64 for 4-items; .82 for 6-items), and slightly higher scores for perceived pros (benefits) of mammography (.82 for 4-items; .80 for 8-items). When survey length is a consideration (e.g., surveillance studies), our results support use of the shorter scales.

A potential study limitation includes the reliance on self-reported outcomes due to the large number of clinics women were navigated to in the urban setting. However, previous validation studies comparing self-reported screenings with medical records show high sensitivity for both mammography (57) and Pap test screening, which increases when recall is within the previous 12 months (58). At follow-up, data collectors and participants were aware of the participants’ study arm due to questions on the follow-up survey related to satisfaction with the intervention. However, we ensured data collectors were not the same individuals as those delivering the intervention to minimize participants’ social desirability bias. Additionally, all participants completed the same structured survey (other than the intervention satisfaction-related questions) and survey protocol process, helping to further minimize differential reporting. Importantly, the strength of using an RCT study design is that it helps to reduce the effect of social desirability and recall bias because the biases are likely to be non-differential across arms, thus helping to minimize systematic differences between intervention and control groups. Another limitation relates to the challenge of contacting women to complete the follow-up surveys. One barrier was the common occurrence of a change in mail addresses (limiting use of mailed reminders to increase participation in follow-up surveys), as well as participants’ temporary loss of cell phone coverage caused by the expense of phone plans. In response, we extended the follow-up period from 6 to 10 months, allowing time for cell phones to be reactivated (after participants could afford to pay their month-to-month phone plan bill). This extended time increased the opportunity for participants to complete the follow-up surveys. Despite challenges in completing the follow-up surveys and differential follow-up with higher rates among the comparison versus intervention arm, the study attrition was non-differential for all demographics, except acculturation level, which was slightly lower among those who participated compared to those lost to follow-up. One reason for this difference may be participation fatigue experienced by intervention participants after receiving multiple calls during the education and navigation program. Intervention participants may have also felt their needs were met and were thus less motivated to engage in the survey, while the comparison group had the opportunity to participate in the program after completing the survey. While acculturation mean scores were overall low for both participants and non-participants, the slightly lower scores among participants may indicate the results are more generalizable to lower acculturated women; however, all participants were low-income and un- or underinsured. While PP analysis is more affected by attrition bias, we conducted ITT analysis, which offers a more conservative estimate of the intervention’s effect by including all participants in the final analysis and assuming no screening for those lost to follow-up. Another limitation was the notable increase in screening rates among the comparison arm, which may be a reflection of the extensive baseline survey (taking an average of 20–40 minutes to complete) having an intervention effect. Regardless, both the mammography and Pap test screening cohorts’ ITT and PP analyses results indicate that the intervention significantly increased screening. An additional limitation relates to the inability to delineate the individual effects of the education and navigation components, as the study was designed to examine the overall intervention effectiveness. Finally, future research is needed to improve the measurement of psychosocial constructs in this population to improve examination of these intermediary factors.

Major strengths of this study are its randomized control trial design, including analysis of effectiveness using ITT analysis. This is important when working with hard-to-reach and vulnerable populations, which can lead to high study attrition rates and biased PP results, yet few CHW-delivered community-based studies report effectiveness based on ITT analysis. Furthermore, our community-based intervention model, bundling mammography and Pap test screening education to deliver to groups of women with different screening needs, followed by one-on-one telephone navigation, offers a scalable strategy to efficiently deliver the program to women in underserved community settings. These findings contribute to the evidence base for CHW-delivered interventions aimed at increasing multiple screenings and reaching large numbers of participants. Our behavioral interventions study also demonstrates the feasibility of CHWs conducting cancer screening needs assessments to recruit primarily uninsured women and to coordinate with community organizations to deliver the program in community settings.

Additionally, in an environment where public health recommendations, such as breast and cervical screening, and HPV vaccination guidelines change over time, the need for effective community-based programs to effectively communicate recommended cancer prevention and control behaviors to women is even more important. Thus, the need for community-based programs, such as the adapted CLS (renamed SEMM) evidence-based program presented in this manuscript, serves as a model for reaching uninsured and underinsured women who may be at risk for not understanding the changing cancer screening and HPV vaccination recommendations.

Ongoing research is needed to evaluate the intervention’s effect on mammography and Pap test screening adherence over time, as well as to evaluate effectiveness on HPV vaccination uptake to further improve breast and cervical cancer early detection and improved outcomes and cervical cancer prevention (1, 59, 60). Additional research could also assess the independent and synergistic effects of group education and one-on-one navigation, for example, through a two-by-two factorial randomized trial, with four study arms [a control arm (delayed intervention), education-only arm, navigation-only arm, and education followed by navigation arm]. This intervention model is currently being adapted and evaluated to reach other underserved populations experiencing similar breast and cervical cancer screening barriers in different geographic regions and among diverse Latina subgroups. Future implementation and dissemination research is needed to support the scale-up of this evidence-based program.

## Data Availability

The datasets presented in this article are not readily available because participant consent did not include public data sharing. Requests to access the aggregated de-identified dataset should be directed to lara.staub@uth.tmc.edu.
